# Effect of Melt Treatment and Heat Treatment on the Performance of Aluminum Cylinder Heads

**DOI:** 10.3390/ma18051024

**Published:** 2025-02-26

**Authors:** Herbert W. Doty, Ehab Samuel, Agnes M. Samuel, Victor Songmene, Fawzy H. Samuel

**Affiliations:** 1 Materials Technology, General Motors Global Technology Center, Warren, MI 48092, USA; herb.doty@gm.com; 2 Département des Sciences Appliquées, Université du Québec à Chicoutimi, Saguenay, QC G7H 2B1, Canada; ehabfhsamuel@gmail.com (E.S.); agnesmsamuel@gmail.com (A.M.S.); 3 Department of Mechanical Engineering, École de Technologie Supérieure, Montreal, QC H3C 1K3, Canada; victor.songmene@etsmtl.ca

**Keywords:** lost foam casting, melt treatment, hardness, tensile properties

## Abstract

The present study was performed on real-life I4-aluminum cylinder heads produced industrially by applying the lost foam technique to Al-Si-Mg alloys (356 and 357). This work, in addition, introduces a new Al-Cu alloys coded 220 alloy. The main aim of this study is to analyze the effects of liquid metal treatment on the hardness and tensile properties of such castings. The effects of liquid metal treatment (modification with 200 ppm Sr, grain refining with 150 ppm B and degassing using pure Ar) of the castings produced by the lost foam technique on the tensile strength and hardness properties were evaluated. Hydrogen plays an important role in the formation of porosity. At the same time, the foam mold leaves an impression on the casting surface taking the shape of fine holes. In addition, segregation of hydrogen occurs in front of the solidification front. Thus, the porosity is a combination of hydrogen level and the solidification rate. Gains of 17% and 24% are observed for the hardness and yield strength for alloy 357 compared to alloy 356, caused by the difference in their magnesium (Mg) contents in the sense that, in the T6 heat-treated condition, precipitates in the form of ultra-fine Mg_2_Si phase particles are formed. The enhancement in the mechanical properties of the used alloy depends mainly of the volume fraction of the precipitated Mg_2_Si particles. The hardness of alloy 220 increases by 18% and the yield strength by 15% compared to that measured for alloy 356. In this case, the hardening phase Al_2_Cu is responsible for this increase. Thus, this study demonstrates that liquid metal treatments significantly enhance the hardness and yield strength of Al-Si-Mg and Al-Cu alloys, with the gain attributed to refined microstructures and reduced porosity.

## 1. Introduction

The first commercial use of the lost foam process was in Germany in 1962 [1,2,3,4]. Initially, it was used in the production of large automotive parts and large-scale parts [5]. Since 1980, several automated production lines with a capacity of 30–60 castings per hour have been in operation. They produce many parts of ferrous and nonferrous alloys. General Motors adopted the lost foam process for the production of its engine cylinder heads in 1981 [6,7]. Saturn Corporation uses the lost foam process to produce most of the aluminum parts for its cars. In 1984, the casting division of the Ford Motor Company launched a fully automated plant for the production of aluminum intake manifolds. In Europe, car manufacturers Citroën, Peugeot, Fiat, Caster (a cast iron foundry), and Alutek (an aluminum foundry) have used the lost foam process on the majority of their production lines [8,9,10].

In Japan, a large steel mill, the Morikawa Sangyo Co. Ltd. (Tokyo, Japan), converted its production operations to the lost foam process in 1984 [11]. Other manufacturers using the lost foam process include the following: in North America—OMC, Mercury Marine, Grede, Quality Aluminum, Ampco, Amcast, Harvard Industries, Mueller Valve Co., and elsewhere in the world—SAFAM, Lucky Gold Star, and others [12]. The outlook is very good for the lost foam process, which could very well be replacing conventional casting methods in the future.

Although aluminum–copper (Al-Cu) alloys have superior mechanical properties to aluminum–silicon (Al-Si) alloys, they are rarely used for lost foam casting. The long solidification time associated with the casting process and the poor formability of Al–Cu alloys are the reasons for this. However, limited research has been conducted on these alloys for lost foam casting [13,14,15].

In the mid-1990s, a second generation of lost foam beads, T180D, which used a blend of three substances as the blowing agent, was marketed for the aluminum market (Table 1). The blend consisted of n-pentane, iso-pentane, and cyclopentane, allowing for the PS beads to be pre-expanded more easily than the first-generation products, T170B, which used only n-pentane as the blowing agent.

Natural aging and sudden increase in mechanical properties occur by the rapid formation of GP (Guinier–Preston) Zones from the supersaturated solid solution and vacancies. The mechanical strength increases remarkably, and the properties become stable after a few hours or even days. Artificial aging involves heating the quenched material to between 95 °C and 205 °C to accelerate the precipitation of certain intermetallic particles in heat-treatable alloys. This acceleration is not entirely caused by the change in the reaction rate. The structural changes are time- and temperature-dependent [16,17].

Precipitation hardening involves heating the alloyed aluminum to temperatures between 100 °C and 230 °C. At these temperatures, the supersaturated solid solution, created by quenching from solution hardening, begins to decompose. Initially, there is agglomeration of solute atoms near the vacancies. Once enough atoms have diffused from their initial positions, a coherent precipitate forms. The precipitate and the aluminum matrix have a lattice mismatch, which results in a stress field around the precipitate. As the solute diffuses into the precipitate, the stress field increases, and eventually the aluminum matrix will no longer be able to accommodate this mismatch. A semi-coherent precipitate will be developed. Eventually, after the semi-coherent precipitate has grown large enough, the matrix can no longer accommodate the crystallographic mismatch, and the equilibrium precipitate will be achieved [17,18,19,20].

Precipitation hardening is the mechanism where hardness, yield strength, and mechanical resistance increase considerably with time at a constant temperature after rapid cooling from a solution temperature. Rapid cooling or quenching following the solutionizing treatment creates the supersaturated solid solution that will provide the “power” for the precipitation. This phenomenon was first discovered by Wilm [21]. He found that the hardness of aluminum alloys with small amounts of copper (Cu), magnesium (Mg), silicon (Si), and iron (Fe) increased with time after quenching a sample at a temperature slightly below the melting point.

For the hardening modulus, the increase in strength is caused by the difference in shear modulus of the matrix and the precipitates [22,23]. In the coherence strengthening mechanism, there is an elastic interaction between the dislocation stress field and the coherent particles. Order strengthening occurs when the precipitate is a superlattice and the matrix is a relatively distorted solid solution [24,25]. Phase decomposition is a special case where the solute concentration in the lattice changes, thus causing a change in elastic strength.

Currently, the most widely used non-ferrous alloys for the lost foam process are undoubtedly from the Al-Si group, mainly alloys 319 and 356 [26,27]. The good fluidity, pressure tightness, and resistance to thermal contractions and shrinkage of Al-Si alloys make them a good choice for lost foam casting. Alloys such as A356 in the T6 condition can have a tensile strength of 228 MPa, a yield strength of 164 MPa, and a percentage strain at fracture of 3.5% [28,29,30]. These mechanical properties are based on tensile specimens cast separately using the sand-casting process. However, there is interest in other alloys that are lighter and have better mechanical properties. The 2XX series alloys fall into this category. These alloys have a tensile strength of 354 MPa, a yield strength of 250 MPa, and a percentage strain at fracture of 7% when sand cast [31].

Thus, while the lost foam process excels in minimizing residual stresses and mold drag forces, its effect on porosity control and hydrogen segregation in Al alloys remains underexplored.

The present work aims at expanding the state of knowledge in the area of manufacturing and optimizing the properties of aluminum products, particularly for the automotive industry. It provides guidelines for changes in the manufacturing process of this type of components through an analysis of the effects of liquid metal processing on the hardness and tensile properties of parts produced industrially from Al-Si-Mg alloys, using the lost foam process. In addition, this work introduces the application of a recent Al-Cu based alloy coded 220 as a new candidate for automotive applications. Also, the present work will allow for technology transfer through a comparison between castings prepared under ideal laboratory conditions and those currently produced in industry in order to improve product quality of the final product. This work stands out from other work conducted on the subject since the parts used for this study are real engine cylinder heads cast in General Motors, USA facilities, unlike others that used small experimental parts produced in laboratories, minimizing certain aspects of the casting technology. This study is expected to bridge the gap by systematically evaluating the influence of melt refinement and alloy composition on microstructure-dependent mechanical properties.

## 2. Experimental Process

In order to extend the scope of the study, three alloys were selected: 356, 357, and 220. In doing so, the parameter specifying the nature of the alloy provided could be isolated. Alloy 356 was chosen since it meets or exceeds all of the requirements that cylinder heads require. Alloy 357 allows for the effects of magnesium concentration to be evaluated since its Mg concentration is 0.6% compared to 0.35% for alloy 356. Experimental alloy 220 differs from other alloys in the 2XX series by its lower copper concentration; Table 2 presents the chemical compositions of these alloys.

**Figure 1:**
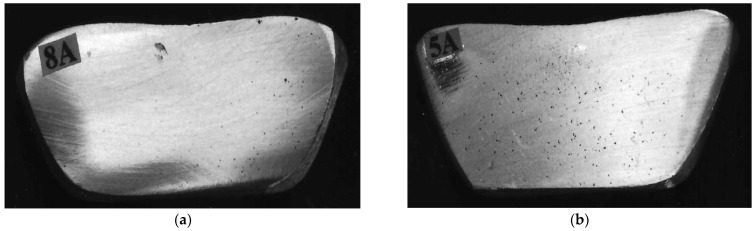
RPT samples corresponding to (**a**) 0.12 mL/100 g Al, (**b**) 0.22 mL/100 g Al H_2_ levels.

The addition of 150 ppm boron ensures good grain refining. The boron (B) master alloy (Al-4% B) was introduced to refine α-Al dendrites, improving homogeneity. Boron was preferred over titanium (Ti) because it provides comparable refining, but requires smaller quantities. The boron concentration in the master alloy is 4%. Degassing for 15 min using pure argon reduced dissolved hydrogen to ~0.12 mL/100 g Al, as verified using AlScan™ technology (Arvida, QC, Canada). A second concentration was obtained by adding a certain amount of hydrocarbon material from raw potato, introducing controlled hydrogen levels for porosity studies, simulating industrial contamination scenarios. The reason for the use of potato is that it is (i) safe (which is an important parameter), (ii) cheap, (iii) does not cause contamination of the melt, and (iv) the evolution of hydrogen gas helps in “cleaning the molten metal”. The hydrogen concentration values were confirmed using a “LECO sub-fusion” analysis-3 samples/condition/alloy. Samples for reduced pressure test (RPT) examination were taken simultaneously, as shown in Figure 1. The four parameters studied are as follows: alloy composition, modification level, refining, and hydrogen content—varied between different levels, as summarized in Table 2. In total, 36 different compositions were tested (Table 3).

**Figure 2:**
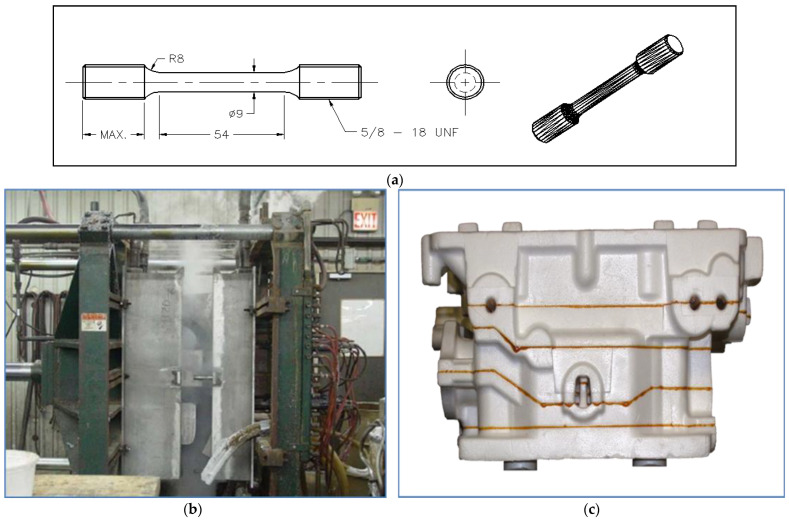

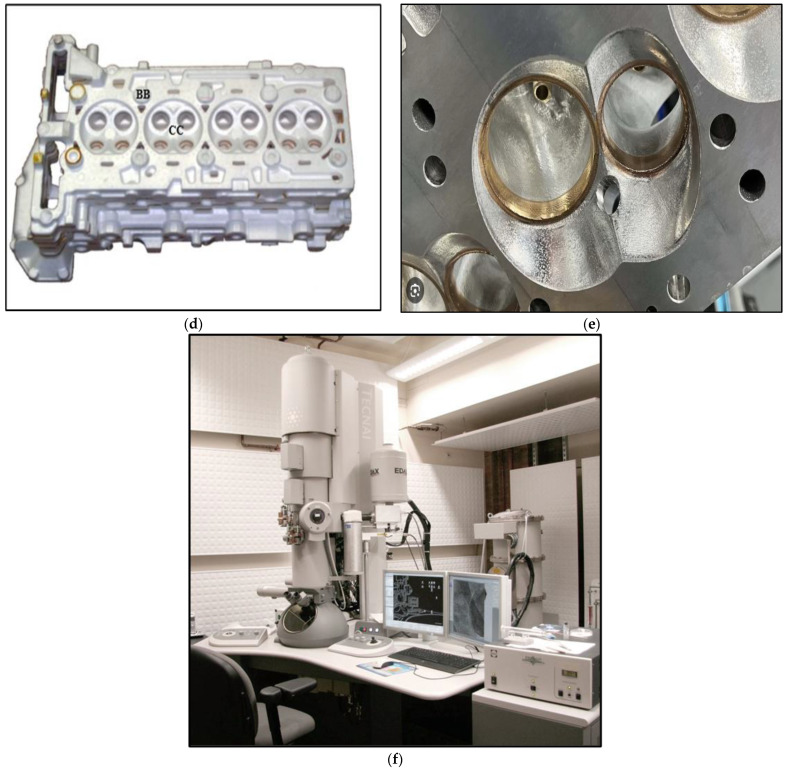
(**a**) Schematic diagram of the LFC tensile test specimen, (**b**) shaping the polystyrene mold, (**c**) assembling the polystyrene mold, (**d**) view of the used cylinder head, (**e**) close-up of GM aluminum cylinder head, (**f**) FEI Tecnai G^2^ F20 Electron Microscope (USA).

Styrochem T-170 balls were used to make the I4 (in-line four-cylinder) cylinder head models. The models were assembled in a cluster and coated with approximately 0.5 mm of Ashland 530 refractory coating using robotic immersion (Figure 2). The models were then set aside to allow for the coating to dry. They were then introduced into a flask, and the free space was filled with unbound lake sand, pushed and compacted by vibration and successive fillings. A ceramic pouring funnel was placed on top of the etching system, and the liquid metal at approximately 800 °C was poured. The liquid metal flowed onto the polystyrene model, replacing the foam. The casting was air-cooled to approximately 150 °C, and the flask was emptied of its contents; the part was separated from the sand, the attack system was removed, and, finally, the part was subjected to heat treatment.

For each of the established conditions (see Table 4), four-cylinder heads are produced. After casting, the cylinder heads are subjected to a T6 heat treatment. Subsequently, four samples are taken: three come from the cylinder head bolt seat or “bolt boss” (BB), two of which will be used to develop tensile test specimens; the fourth sample comes from the combustion chamber (CC) and will be used for metallographic analysis. Normally, the seat of the cylinder head bolts is pierced with a hole, allowing for the passage of the bolts towards the block where they will be fixed; however, this hole is only drilled during final machining, so the sample from this location is a solid part and does not have any holes.

The samples collected are cut, crimped, roughly ground, and then polished according to an established procedure reported in Table 4. Subsequently, the interdendritic distance, eutectic silicon particle size, porosity, pore size, and grain size (ASTM E 112) are measured and analyzed using a Leica optical microscope coupled with a Clemex image analysis system at different magnifications (50× to 500×). The measurements are carried out on 30–50 fields, depending on the magnification, in order to systematically cover the entire surface. A chemical attack is necessary for the measurement of the grain size; the samples are immersed for about one minute in a solution containing 66% vol. nitric acid (concentration of 68% vol.), 33% vol. hydrochloric acid (concentration of 35% vol.), and 1% vol. hydrofluoric acid (concentration of 48% vol.). Figure 2b exhibits a part of the process of making a polystyrene model of four-cylinder heads.

The heat treatment applied to samples produced in the laboratory aims to improve their mechanical properties. It is systematically applied to all samples before analysis. The chosen heat treatment, T6, consists of solution treatment followed by quenching and artificial aging. Table 5 shows the different treatment parameters according to the alloy.

Except for the parts produced using the standard mold, which already have the final shape of tensile specimens, the other parts are machined to produce tensile specimens. Their shape differs slightly because there is not enough material available to make bars of the same size. However, the dimensions of these specimens comply with the ASTM E8-04 standard. Tensile tests (ASTM B557120) were performed using a 100 kN MTS servo-hydraulic system. The strain rate used was 8.3 × 10^−4^ s^−1^. An attachable extensometer (strain gauge was used to measure the deformation that takes place in the samples during the test, and the data acquisition system attached to the machine converts it to an accurate measure of the percentage elongation. The stress required for rupture (UTS), the elastic limit (YS), and the percentage of deformation (% Elongation) were the measured parameters. Brinell hardness tests were performed according to ASTM E10121, 10 mm ball, 500 kg load for 30 s. The indentations were made on the tensile specimens machined to create two parallel surfaces. Six prints were taken per sample.

In addition to optical metallographic analyses and mechanical tests, samples were subjected to other analyses: scanning electron microscopy (SEM), wavelength dispersive spectrometry (WDS), energy dispersive spectrometry (EDS), electron probe microanalysis (EPMA), and radiography. These analyses allow for the evaluation of the phases present in the samples, their chemical composition, as well as a visualization of the porosity inside them. Scanning electron microscopy is a powerful technique for observing samples. It is mainly based on the detection of backscattered electrons emerging from the surface under the impact of a very fine beam of primary electrons that scans the observed surface and allows images to be obtained with a resolving power often less than 5 nm and a large depth of field.

**Figure 3:**
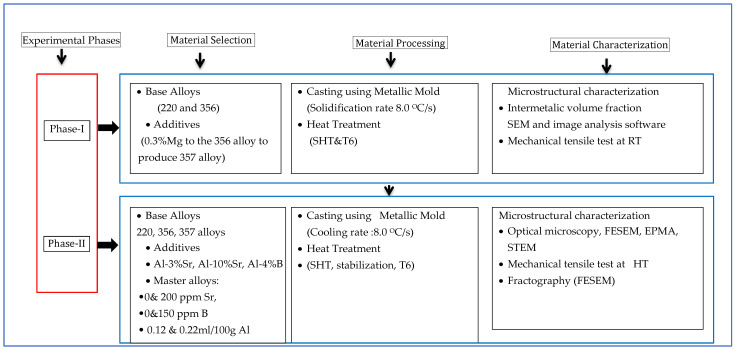
Summary of experimental plan.

An electron probe microanalyzer operates by reducing an electron source by two or three magnetic lenses. The electrons strike the sample to be analyzed with an impact energy that can vary from a few hundred eV to 50 keV. The X-rays emitted by the sample under the impact of the electrons are analyzed using X-ray spectrometers that can be of the WDS (wavelength dispersion) or EDS (energy dispersion) type. The system generally uses the transition lines to the K electronic level, because these are the lines that separate most effectively. The excitation mode of the atoms makes it possible to draw up a chemical map of the sample. Selected samples were examined using transmission electron microscopy to investigate the coherency of the precipitates with the matrix. The FEI Tecnai G2 F20 electron microscope (Hillsboro, OR, USA),employed is equipped with an advanced control system, which permits the integration of an EDAX™ chemical analysis system, scanning transmission electron microscopy (STEM), and electron energy loss spectroscopy (EELS). The microscope was operated at an accelerating voltage of 200 kV—Figure 2f. Figure 3 summarizes the main outlines of the adopted work plan.

## 3. Results and Discussion

The mechanical properties of a casting are a reflection of its quality. There are several tests that can be used to assess the performance of alloys, the most common of which are undoubtedly the tensile test and the hardness test. The quality index is not a mechanical test to assess the performance of alloys, but rather a tool that uses the results from other mechanical tests and mathematical equations to assess the quality of alloys.

The secondary dendrite arm spacing (SDAS) for samples from the lost foam process varies depending on the location of the sample taken or the wall thickness. The specimens used for the tests all come from the same location (cylinder head bolt seat), and the section size is also identical. The average value of the SDAS measured for this location is 68 µm, which is relatively high and suggests lower mechanical properties.

### 3.1. Effect of Alloy Composition

**Figure 4:**
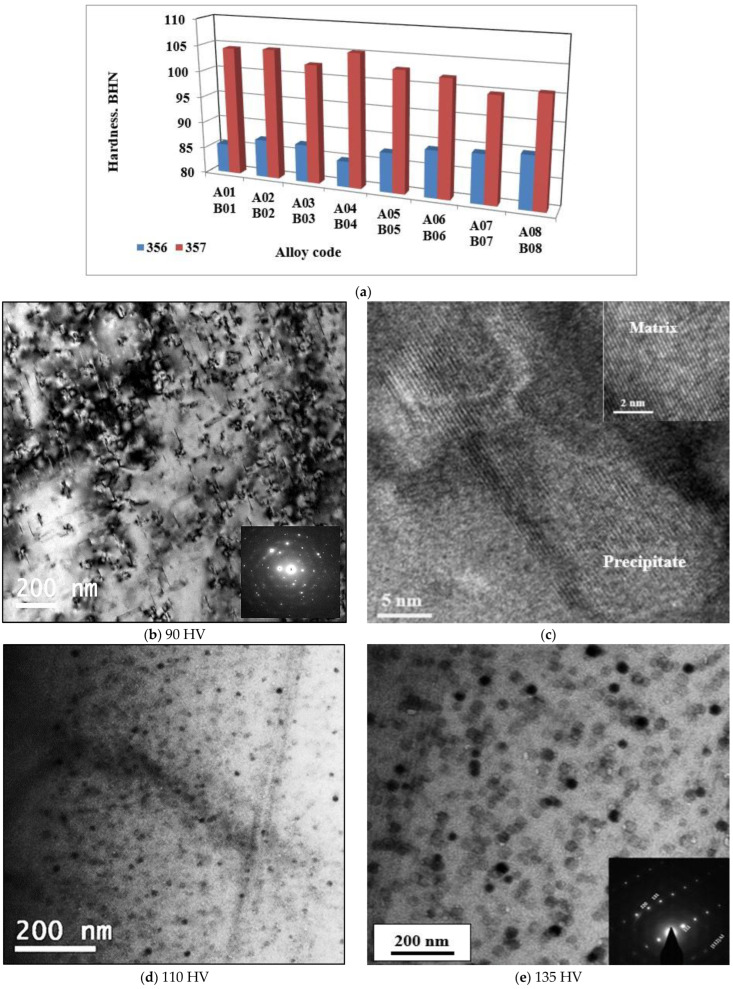
(**a**) Brinell hardness of samples of alloys 356 and 357 cast using the lost foam process; High resolution electon images showing precipitation of Mg_2_Si phase particles in B01 alloy after aging at: (**b**,**c**) 155 °C, (**d**) 170 °C, (**e**) 190 °C. Respective Vickers hardness values of the samples shown in (**b**,**d**,**e**) are mentioned below the figures.

It has been shown that, at the microstructural level, alloys 356 and 357 respond to liquid metal treatments in almost the same way; the differences observed between these two alloys are negligible, with a few exceptions, such as the formation of Mg_2_Si. However, this small difference has a major impact on the mechanical properties of the samples from these alloys produced using the lost foam process. The samples used to perform the hardness tests were also taken from the seat of the cylinder head bolts. The variation in hardness within the same alloy, whether 356 or 357, is negligible. Regardless of the condition used, the average hardness value remains approximately the same, 88 BHN for alloy 356 and 103 BHN for alloy 357 (Figure 4), which represents an improvement of almost 17%. The addition of boron, strontium, or hydrogen has no significant effect on the hardness value. Moreover, statistical analysis supports this hypothesis, which makes the nature of the alloy, by itself, responsible for the hardness of the samples.

Metallographic examination demonstrated the presence of Mg_2_Si phase particles for alloys 356 and 357. The precipitation of this phase during T6 heat treatment is responsible for the hardening of the alloys. Since the magnesium concentration of alloy 357 is twice as high as that of alloy 356, the amount of Mg_2_Si phase particles is higher. This results in a more pronounced improvement in hardness for alloy 357 compared to alloy 356. The precipitation sequence of these particles begins with the formation of GP zones followed by the metastable β′ phase (Mg_2_Si) and, finally, the equilibrium β phase (Mg_2_Si). The GP zones are needles oriented in the {001} direction; moreover, β′ and β show a similar orientation.

In order to follow on the above-mentioned precipitation sequences, some samples were re-solutionized, quenched, and aged at 155 °C, 170 °C, and 190 °C for 4 h each, followed by air-cooling. The samples were examined using HR-STEM, operating at an accelerating voltage of 200 kV. Hardness was measured using a microhardness tester (100 g).

Figure 4b shows a TEM bright field image revealing the precipitation of GP zones surrounded by high contrast of the aluminum matrix. In this case, the electron diffraction pattern shows un-identified scattered spots. However, in Figure 4c, a high-resolution image corresponding to Figure 4b, displaying a full coherency between the precipitates and matrix–interplanar directions of both the matrix and precipitate are the same. When increasing the aging temperature to 170 °C, the bright field shown in Figure 4d exhibits fine dispersed round particles. With further increases in the aging temperature (present T6 temper), the microstructure reveals a significant coarsening of the precipitated particles with an electron zone axis close to [112] direction, Figure 4e.

**Figure 5:**
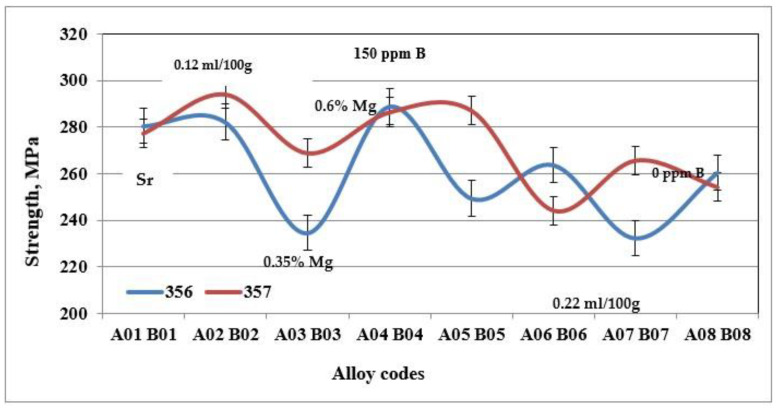
Ultimate tensile strength for specimens of alloys 356 and 357 cast using the lost foam process.

**Figure 6:**
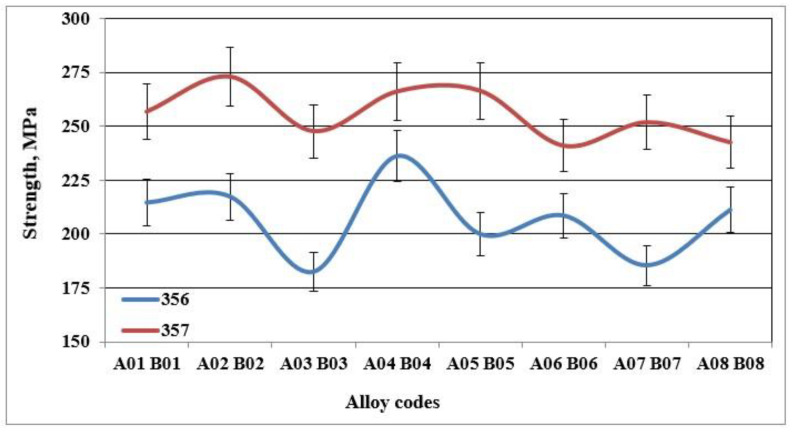
Yield strength of specimens of alloys 356 and 357 cast using the lost foam process.

**Figure 7:**
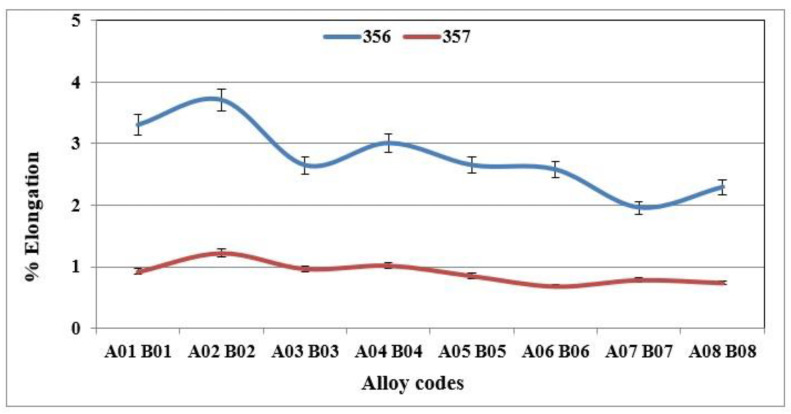
% Elongation to fracture of specimens of alloys 356 and 357 cast using the lost foam process.

Figure 5, Figure 6 and Figure 7 show the results obtained during tensile tests for samples of alloy 356 produced using the lost foam process. The average value of the ultimate tensile strength is 262 MPa, while that of the yield strength is 207 MPa. Despite a relatively high SDAS, these values remain excellent compared to the standard results (SDAS 25 µm), which are 262 and 185 MPa, respectively. The major difference appears at the level of ductility, which seems to be greatly affected by the lost foam process. The average value of ductility observed for samples of alloy 356 produced using the lost foam process is 2.8% compared to 5%, a value normally measured in standardized samples.

The behavior of alloy 357 is different from that of alloy 356, since the same precipitated particles, i.e., Mg_2_Si, responsible for the increase in hardness of the samples of alloy 357 are also responsible for the improvements in their tensile mechanical properties. Figure 6 shows that the values of the ultimate tensile strength for alloy 357 are 273 MPa, an improvement of 4% compared to alloy 356. The improvement in the mechanical properties becomes clearly evident for the yield strength, which amounts to 256 MPa, which represents an increase of 24% compared to alloy 356. The formation of particles of the Mg_2_Si phase has a direct consequence, especially after the application of the T6 type heat treatment, in hardening of the alloys, which leads, however, to a decrease in ductility. Samples of alloy 357 behave similarly, which explains the ductility values depicted in Figure 7.

### 3.2. Effect of the Strontium Concentration of the Modifying Agent

**Figure 8:**
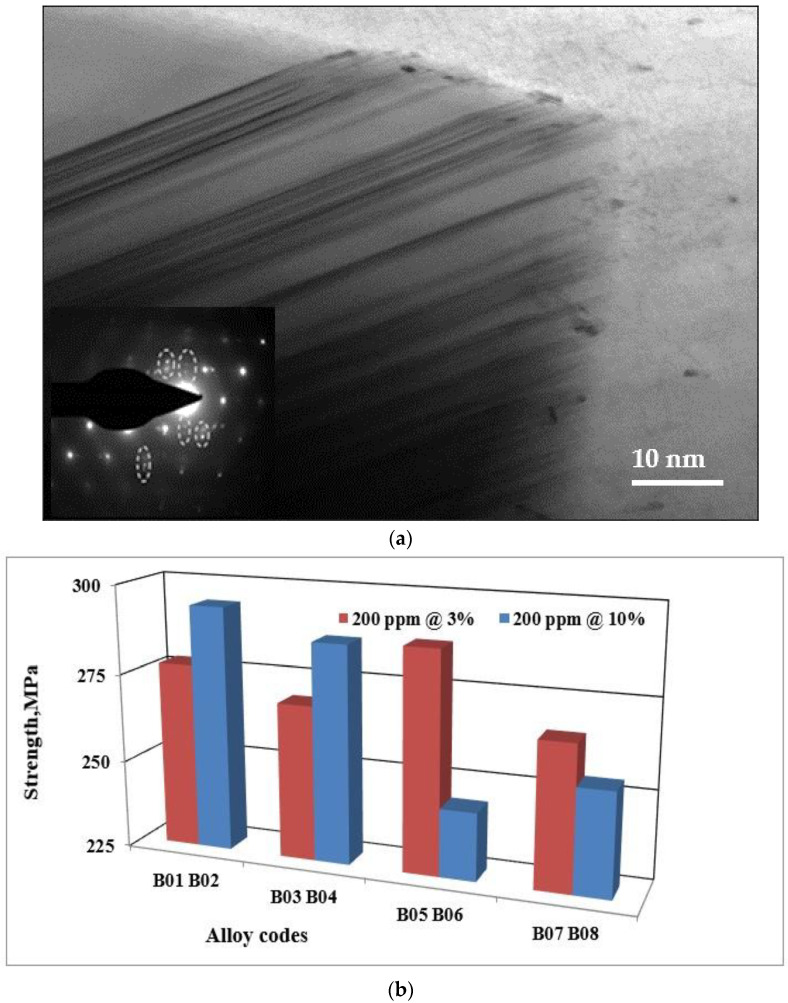
(**a**) Twinning in 357 alloy modified with 200 ppm Sr (Al-Sr master alloy). Note the presence of double spots in the electron diffraction pattern outlined by the broken white circles. (**b**) Ultimate tensile strength of alloy 357 samples obtained from the lost foam process as a function of the strontium master alloy concentration.

The flake-like Si is can be changed into a fibrous morphology by a process called modification. This operation (modification) is achieved by in several ways; the most common ways are chemical modification and quenching modification. The former can be produced by adding transition elements such as strontium (Sr) [29]. Two theories were used to explain the chemical modification effect: the restricted nucleation theory and the restricted growth theory [30]. According to restricted growth theory, the impurity induced twinning impairs the Si growth by poisoning the growing Si ledges, stopping the twin plane re-entrant mechanism, and explaining the twin plane re-entrant edge (TPRE) poisoning [31]. Figure 8a demonstrates the growth in eutectic Si particles in Sr-modified Al-Si-Mg alloys, revealing intense twinning.

The use of strontium master alloys of different concentrations has no effect on the hardness value. Furthermore, the variations observed for the ultimate tensile strength, the yield strength, and the ductility when adding these master alloys are not significant. The difference between the various values is less than that in the standard deviation for these values. The plot in Figure 8b demonstrates that the variation in the values of the ultimate tensile strength for alloy 357 is not significant. These observations are also valid for alloy 356.

### 3.3. Effect of Boron Grain Refining

**Figure 9:**
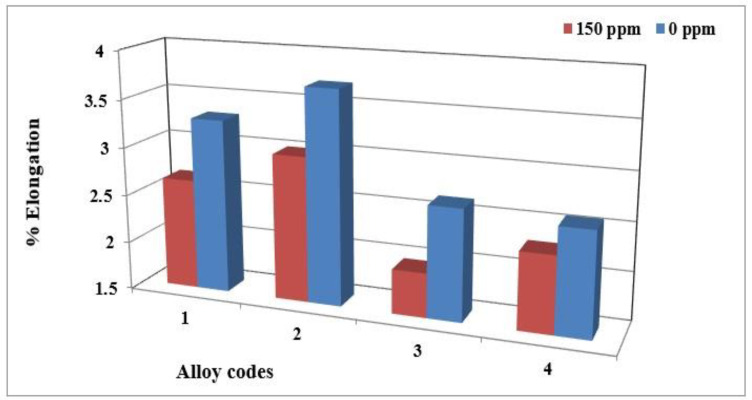
Elongation to fracture of alloy 356 samples from the lost foam process as a function of boron concentration.

Using 150 ppm boron in the form of a Al-4%B master alloy was found to have no significant effect on the hardness value. Moreover, no changes were observed for the ultimate tensile strength and yield strength. However, its effects on elongation are significant. Adding boron to the base alloy decreases the ductility of alloy 356 samples by up to 20% (Figure 9). This decrease is caused by the improvement in the porosity distribution rather than by the decrease in grain size [32]. The behavior of alloy 357 is slightly different since the effects of refining on ductility are minimized by the hardening effect provided by the Mg_2_Si phase particles. The results obtained demonstrate that the average value of ductility for samples of alloy 357 produced using the lost foam process hardly varies with the amount of refining agent added.

**Figure 10:**
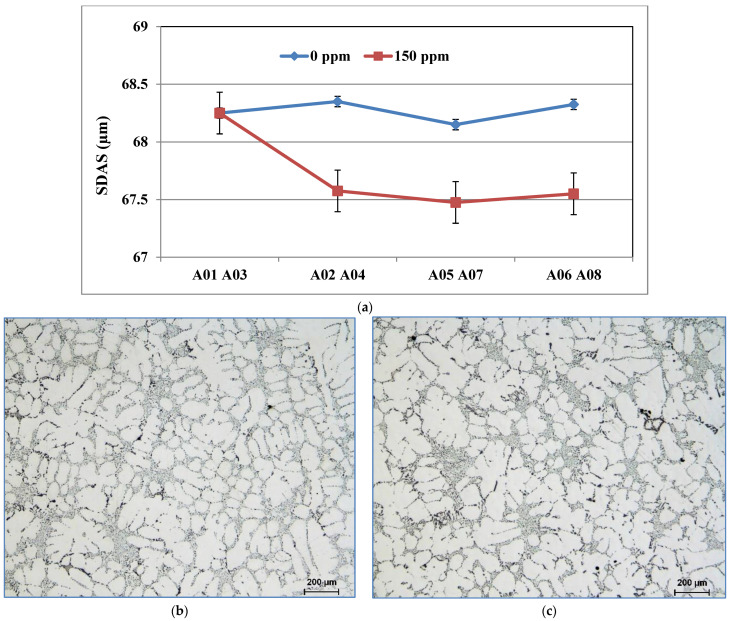
(**a**) Secondary dendrite arm spacing of cylinder head bolt seat samples for alloy 356 cast using the LFC process as a function of boron refining. Morphologies of the dendritic α-Al phase of alloy 356 cast samples using the lost foam process: (**b**) A03 and (**c**) A04 (150 ppm B).

During solidification, the AlB_2_ particles are pushed into the interdendritic regions and do not act directly on the nucleation of the α-Al phase. On the other hand, the silicon of the 356 or 357 alloys present in the dendritic region precipitates on the AlB_2_ particles, which will subsequently act as nucleation sites for the formation of the α-Al phase. The addition of boron in the form of Al-4%B master alloy does not affect the value of the SDAS (Figure 10a); however, it changes the morphology of the α-Al dendritic phase. The samples from the unrefined conditions have an elongated shape with a generally non-uniform microstructure, Figure 10b, while the samples from the refined conditions have a rounded structure, Figure 10c. The volume fraction of the α-Al phase remains unchanged regardless of the shape that the dendrites will take. The same observations are also made in the samples of alloy 357.

Grain refining is a result of two separate processes: nucleation of new crystals from the melt, followed by growth with a limited size. Both processes need a driving force that must be provided to the system through supercooling and supersaturation compared to the equilibrium conditions of the real system. During the entire first period of the solidification process, only those parts of the liquid metal that are in contact with the mold walls are solidified to such an extent that the nucleation of new aluminum grains can occur. It was proven that nucleation starts above the steady-state growth temperature [33]. This means that new crystals can be formed not only at the first contact of the melt with the cold mold walls, but also in the liquid.

**Figure 11:**
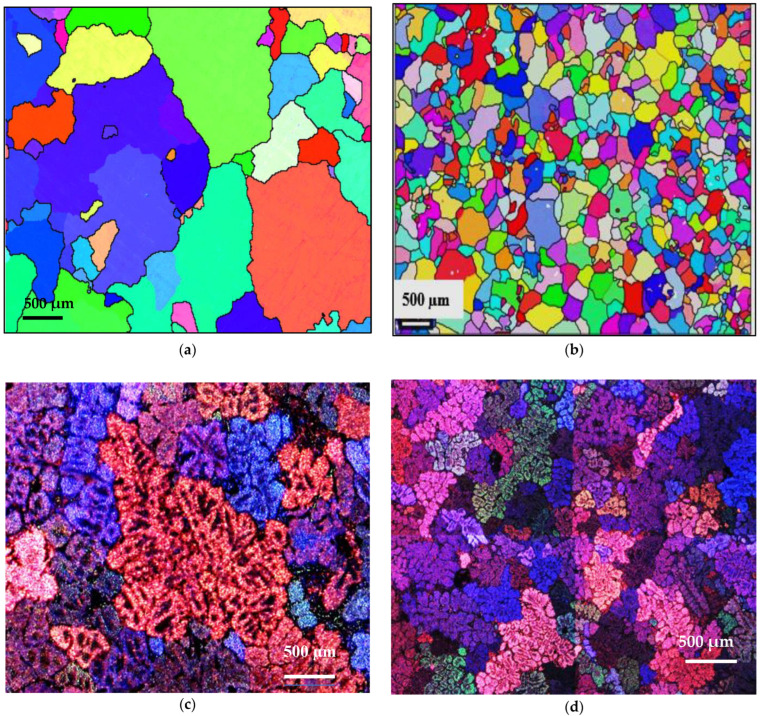

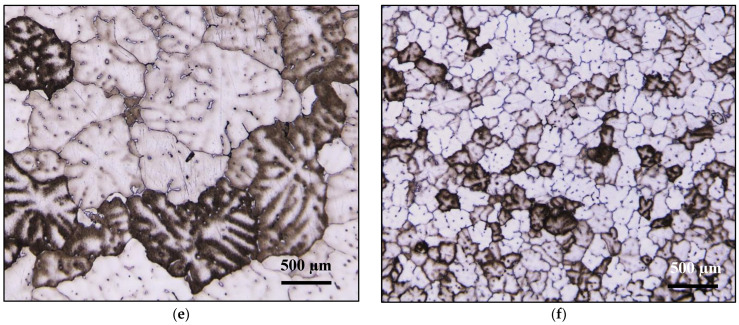
Effect of B addition on grain-refining of Al-Si-Mg and Al Cu-Mg alloys in samples: (**a**,**c**) A11, (**b**,**d**) A12, (**e**) C9, (**f**) C10.

Grain refining has a great influence on mechanical properties, since these are improved by reducing the grain size, i.e., through grain boundary strengthening. For some metals and alloys, the yield strength is inversely proportional to the grain size, which is determined by the Hall–Petch relation [34]: Re_0.2_ = σ_0_ + 1/k√d, where Re_0.2_ is the conventional yield strength, σ_0_ is a constant whose dimensions are those of a stress, k is a parameter whose value depends on the material, and d is the average grain size. Figure 11 reveals the refining effectiveness when added to the three studied alloys, regardless of their chemical composition. However, the degree of refining depends on the presence of traces of Ti in the base alloy, leading to the formation of TiB_2_. Thus, in this case, grain refining will take place through both TiB_2_ and AlB_2_ particles.

### 3.4. Effect of Hydrogen

**Figure 12:**
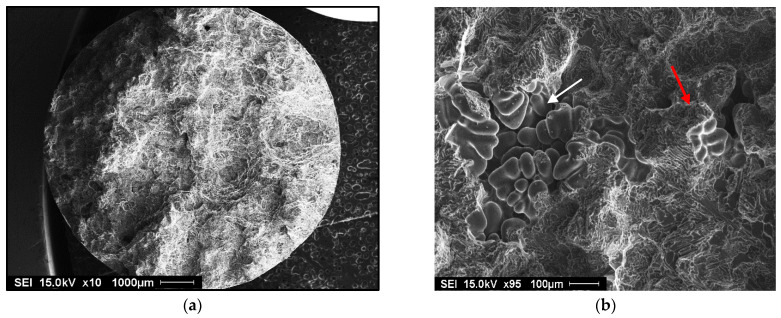

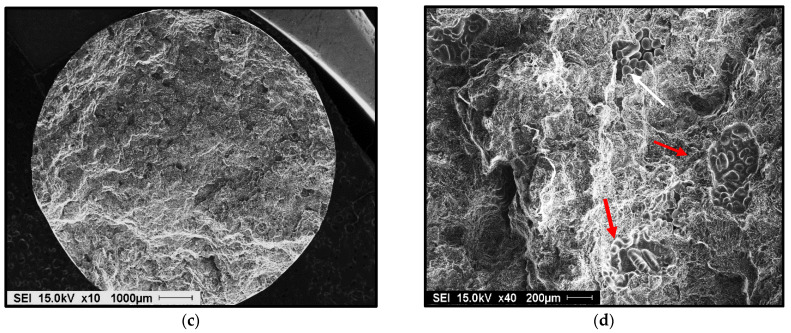
Porosity formation in 356 alloy containing: (**a**) 0.12 mL/100g Al H2 - general view, (**b**) high magnification of (**a**) showing the presence of gas and shrinkage porosity, (**c**) 0.22 mL/100 g Al H2 - general view, (**d**) presence in gas porosity in (**c**). Red arrows indicate gas porosity, whereas white arrows point to shrinkage porosity.

Germination is the process by which hydrogen bubbles concentrate in the interdendritic liquid. If their energy is sufficient, they increase in volume, but they remain trapped in the pasty zone. After solidification, the porosity volume increases slightly due to solidification shrinkage [35,36]. Examples of gas and shrinkage porosity are shown in Figure 12.

**Figure 13:**
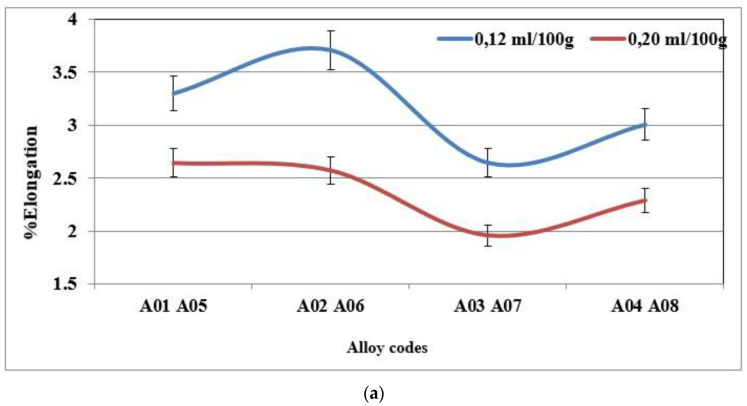

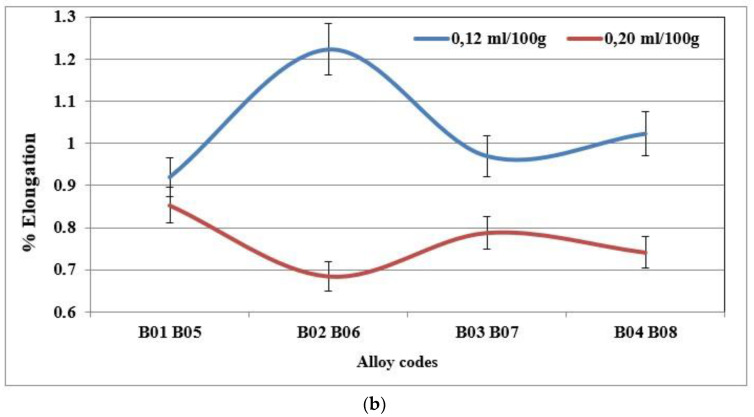
(**a**) % Elongation to fracture for specimens of alloy 356 cast using the lost foam process as a function of hydrogen level (SDAS 68 µm-BB). (**b**) % Elongation to fracture for specimens of alloy 357 cast using the lost foam process as a function of hydrogen level (SDAS 68 µm).

The addition of hydrogen has no effect on the hardness value, the ultimate tensile strength or the yield strength. However, the ductility decreases when the hydrogen level in the alloy is high. The plots presented in Figure 13a,b display the percent elongation to fracture of the specimens of alloys 356 and 357 cast using the lost foam process, as a function of the hydrogen level. They highlight the loss of ductility for the high hydrogen concentration. For alloy 356, the loss of ductility is of the order of 25% between the low and high concentration [18,37,38,39]. 

**Figure 14:**
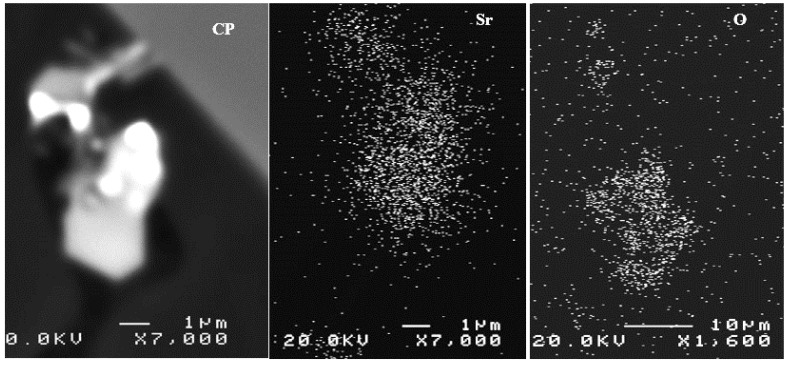
Example of SrO observed in the B07 alloy.

Hydrogen in the liquid metal appears to be the source of the loss of ductility incurred in castings produced using the lost foam process. Pores caused by hydrogen (or hydrogen-induced porosity) embrittle the samples by facilitating the decohesion of the metal. The embrittlement of aluminum alloys depends on the microstructure, strain rate, and temperature. Generally, under-aged alloys are more prone to embrittlement than over-aged ones. Another parameter to consider is the formation of SrO during the course of melting and casting (A07 and B05 for example). Figure 14 depicts examples of SrO observed in Sr-treated samples [40,41,42].

**Figure 15:**
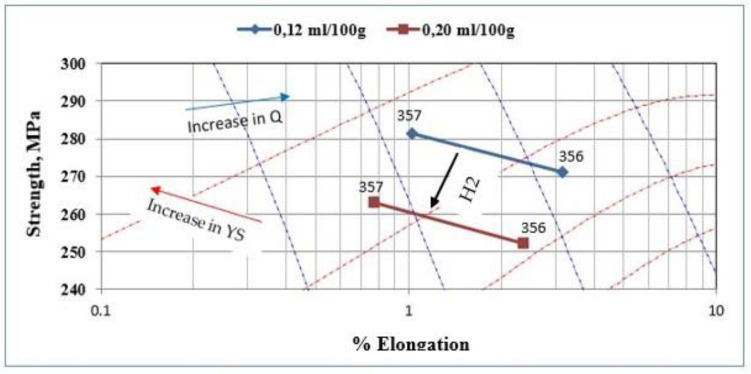
Quality index of the samples of alloys 356 and 357 produced using the lost foam process as a function of hydrogen level. The blue dashed lines and the red dashed lines represent the iso-Q and iso-YS lines in the chart. The black arrow shows how the strength changes with the hydrogen content of the samples [46].

The correlation between the quality index measurement and explanatory variables, such as the nature of the alloy used, the added boron content, the type of strontium master alloy used, or the measured hydrogen level, is rather weak. In fact, only the hydrogen level seems to have a significant effect on the quality index value. This effect remains moderate and does not alone fully explain the behavior of the quality index. Figure 15 shows the plot of the quality index of the samples of alloys 356 and 357 produced using the lost foam process as a function of the hydrogen level present in these samples. The alloy 356 seems to have an advantage over alloy 357 in terms of quality index; however, the latter displays a better yield strength. As mentioned previously, the hydrogen level affects the quality index value; a decrease of 11% is observed for alloy 356, while alloy 357 shows a decrease in the order of 13% when hydrogen is present in a high concentration (0.22 mL/100 g Al) [43,44,45].

**Figure 16:**
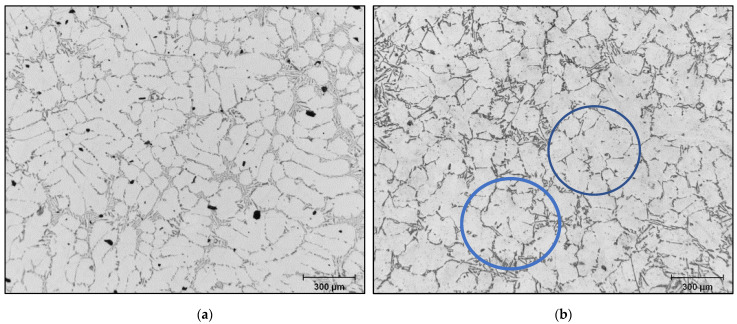
Optical microstructure of 356 alloy: (**a**) no B, (**b**) 150 ppm B-blue circles showing the change in the shape of the dendrites from elongated into rounded ones.

When the B-refiner is added to the liquid metal, the dendrites of the α-Al phase take on a rounded form (Figure 16). This change in the dendrite morphology results in a decrease in the apparent viscosity of the alloy. For all additions of grain refiner, the volume fraction of the α-Al dendritic phase appears unchanged. Grain refining plays an important role in the deformability of alloys by reducing the surface tension of the residual liquid, which also reduces the apparent viscosity of these alloys. This is supported by the results obtained by [47,48,49].

### 3.5. Mechansim of Grain Refining with B

**Figure 17:**
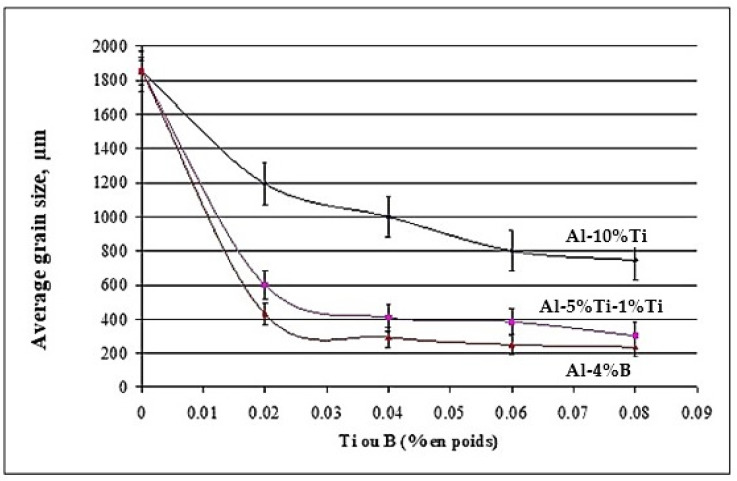
Grain refining of alloy 356 with different master alloys.

The effect observed as a result of the addition of titanium and low boron content in the hypoeutectic alloy A356 is consistent with that obtained by [50,51]. Indeed, the grain size rises to 1854 µm in the base alloy in the absence of any addition. The performance of the three grain refiners is presented in Figure 17. The binary alloy Al-10%Ti proves to be less efficient, since the grain size stabilizes approximately at 800 µm, while the boron master alloy (Al-4%B) is more powerful in the hypoeutectic Al-Si alloys, since the size drops to reach a minimum value of the order of 250 µm. At a certain level, the grain size remains constant, even if the added amount of Ti and B increases, hence the plateau observed in the curve of Figure 17.

The thermal analysis results for the Al-7%Si 356 alloy clearly show that solidification of this alloy requires a supercooling ΔT = TN2 − TN1 in the order of 1.90 °C in the absence of the grain refiner. However, the addition of 0.02%Ti (Al-10%Ti) and 0.02%B (Al-4%B) resulted in reducing this supercooling to 1.12 and 0.75 °C, respectively. The low or almost zero undercooling observed after the addition of 0.02%B clearly reveals that the Al-4%B master alloy results in better grain improvement in the case of the Al-7%Si alloy when compared to the Al-10%Ti and Al-5%Ti-1%B master alloys at a similar level of addition. This supports the results of from the optical macroscopy and microscopy observations [52].

**Figure 18:**
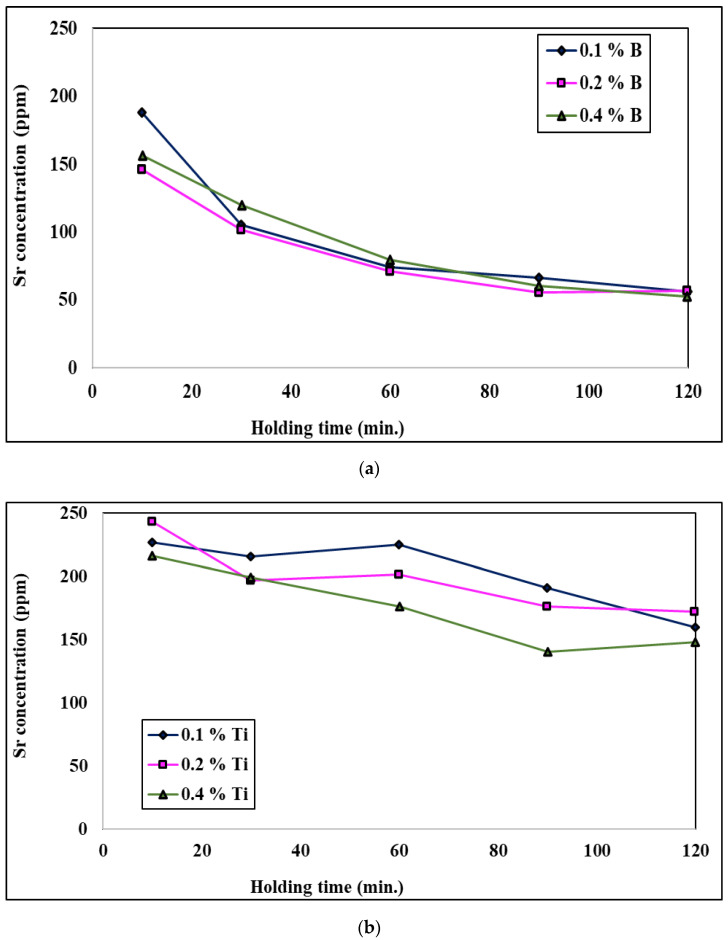
Effect of the addition of (**a**) B and (**b**) Ti on the remaining Sr in a molten aluminum bath.

When the molten bath is treated with both a grain refiner and a modifier, the evolution of the Sr concentration in the molten metal is highly time-dependent for higher levels of addition of the Al-Ti-B grain refiners; Figure 18 shows this phenomenon. The zero-time concentration is the Sr level in the molten aluminum before the addition of either of the Al-Ti-B grain refiners. After addition, a weakening of Sr is observed for both molten alloys; the molten bath treated with the Al-1.5Ti-1.5B master alloy loses its Sr much more rapidly, especially at the initial stage after addition, compared to the molten bath treated with Al-5Ti-1B. This explains the rapid loss in eutectic modification in the alloy treated with Al-1.5Ti-1.5B, meaning that there is insufficient free Sr in the molten aluminum to modify all of the eutectic silicon. The rapid loss in strontium can be explained by external oxidation and vaporization [26,53,54,55,56,57,58].

## 4. Conclusions

The study of the effects of liquid metal treatment on the microstructure of samples of alloys 356 and 357 produced by the lost foam casting process was carried out using several analytical techniques: optical microscopy, image analysis, and scanning electron microscopy. The effects on mechanical properties were also evaluated through a comprehensive analysis of tensile and hardness tests carried out on specimens of these alloys. In light of the results obtained, the following conclusions can be drawn:

1. The values of the ultimate and yield strengths of the samples from the lost foam process are consistent with the mechanical properties presented in most reference works for the same secondary dendritic arm spacing; however, the value of ductility is reduced by half by the porosity observed in the LFC samples.
2. The values of hardness and yield strength vary according to the chemical composition of the alloy used. Gains of 17% and 24% are observed for hardness and the yield strength for alloy 357 compared to alloy 356. This difference is caused by the different concentrations of magnesium which, under the action of the T6 heat treatment, precipitate in the form of Mg_2_Si.
3. The hardness of alloy 220 increases by 18% and the yield strength by 15% compared to that measured for alloy 356. In this case, the hardening phase Al_2_Cu is responsible for this increase. All these increases in the hardness value are independent of the type of mold used. In addition, the hardness value is affected by the solidification rate.
4. No effect was observed with the concentration of strontium in the master alloy used for modification i.e., Al-3%Sr or Al-10%Sr, on the mechanical properties of samples from any type of molds.
5. The addition of 150 ppm boron decreases the ductility of samples of alloy 356 by 23% and increases the ductility of samples of alloy 220 by 150%.
6. The properties of alloy 356 are controlled by the eutectic, and any influence on grain size per se is secondary. However, for alloy 220, the grain size is particularly important for the distribution of intermetallic particles, since grain refining redistributes these particles in a more favorable manner.
7. The study confirms that Sr modification, B refining, and degassing reduce SDAS and porosity, yielding superior mechanical properties. These findings can provide useful information in the production of high-strength automotive components using lost foam casting.
8. Boron-treated samples exhibited increased nucleation density of the α-Al grains, reducing SDAS and enhancing yield strength by ~15%.

### Suggested Ideas for Future Work

Since the local solidification time is largely influenced by the filling and residue removal processes involved during casting using the lost foam process, an in-depth study of samples from different locations in the casting would provide a better understanding of how the geometry of the mold, the type of sand used, and the nature of the lost foam model act on the local solidification time and, consequently, on the mechanical properties. 

In addition, the lost foam technique seems very sensitive to casting parameters, so the use of Al-Si-Cu or Al-Cu alloys, such as alloy 319 or 206, would make it possible to increase our knowledge and study the potential of lost foam casting to make parts from other commercial alloys normally used in proven shaping techniques such as die casting or sand casting.

A study could also be carried out on developing mathematical models describing the effects of the process parameters on the analyzed responses, as well as statistical analysis, including ANOVA analysis.

## Figures and Tables

**Table 1:** First generation of commercial-grade expanded polystyrene (EPS) beads for lost foam [15].

Product	Average Beads Size (μm)	Potential Density (Kg/m^3^)	Weight of Blowing Agent (%)	n-Pentane (%)	Iso-Pentane (%)
T170B	350	24.0	5.7–6.4	100	0
T180D	350	17.6	6.2–7.0	70	15
X180	250	20.8	6.2–7.0	70	15
D180B	450	16.0	6.2–7.0	70	15

**Table 2:** Composition of the different alloys used as received (wt.%).

Alloy	Si	Cu	Mg	Fe	Mn	Zn	Ti	Sr	Al
220	1.32	2.09	0.42	0.58	0.60	0.00	0.00	0.00	bal.
356	6.78	0.02	0.35	0.11	0.04	0.04	0.08	0.00	bal.
357	6.78	0.02	0.60	0.11	0.04	0.04	0.08	0.00	bal.

**Table 3:** Variables applied for experimental castings.

Variable	Level 1	Level 2	level 3
A: Alloy composition *	220 Al-2%Cu-0.4%Mg	356 Al-7%Si-0.35%Mg	357 Al-7%Si-0.6%Mg
B: Al-Sr master alloys; Sr (ppm)	0	200 (Al-3%Sr)	200 ppm (Al-10%Sr)
C: Boron (ppm)	0	150	-
D: Hydrogen (mL H_2_/100 g Al)	0.12	0.20	-

* See Table 2 for complete chemical compositions.

**Table 4:** Codes of the used castings.

Code	Alloy	Condition		
		Al-Sr (%) Master Alloy (200 ppm) *	Boron (ppm)	H_2_ (mL/100 g)
A01	356	3 *	0	0.12
A02	356	10 *	0	0.12
A03	356	3 *	150	0.12
A04	356	10 *	150	0.12
A05	356	3 *	0	0.20
A06	356	10 *	0	0.20
A07	356	3 *	150	0.20
A08	356	10 *	150	0.20
A09	356	0	0	0.12
A10	356	0	150	0.12
A11	356	0	0	0.20
A12	356	0	150	0.20
B01	357	3 *	0	0.12
B02	357	10 *	0	0.12
B03	357	3 *	150	0.12
B04	357	10 *	150	0.12
B05	357	3 *	0	0.20
B06	357	10 *	0	0.20
B07	357	3 *	150	0.20
B08	357	10 *	150	0.20
B09	357	0	0	0.12
B10	357	0	150	0.12
B11	357	0	0	0.20
B12	357	0	150	0.20
C01	220	3 *	0	0.12
C02	220	10 *	0	0.12
C03	220	3 *	150	0.12
C04	220	10 *	150	0.12
C05	220	3 *	0	0.20
C06	220	10 *	0	0.20
C07	220	3 *	150	0.20
C08	220	10 *	150	0.20
C09	220	0	0	0.12
C10	220	0	150	0.12
C11	220	0	0	0.20
C12	220	0	150	0.20

* 200 ppm Sr/casting.

**Table 5:** Description of the T6 treatment steps for the different alloys studied.

Step	220	356	357
Solutionizing treatment	5 h @490 °C	5 h @540 °C	5 h @540 °C
Quenching	Water @60 °C	Water @60 °C	Water @60 °C
Stabilization	-	24 h @20 °C	24 h @20 °C
Aging	4 h @190 °C	4 h @160 °C	3 h @190 °C

## Data Availability

The original contributions presented in this study are included in the article. Further inquiries can be directed to the corresponding author.

## References

[1] Shroyer H.F. (1958). Cavityless Casting Mold and Method of Making Same. U.S. Patent.

[2] Ambert P., Barge H., Bourhis J.R., Esperou J. (1985). Mise en évidence, âge et niveau technique des exploitations préhistoriques cuprifères de Cabrières (Hérault). Archéol. Languedoc.

[3] Barron J.H. (1965). Full Mold Process-the Direct Approach to Metal Casting. AFS Trans..

[4] Dieter H.B., Paoli A.J. (1967). Sand Without Binder for Making Full Mold Castings. AFS Trans..

[5] McElroy J. (1982). Lost Foam Casting Breaks Into High Volume. Automot. Ind..

[6] Mullins P.J. (1984). Adhesives Europe—The New Ties That Bind. Automot. Ind..

[7] Lincoln M. (1984). Lost Foam Finds New Applications. Mach. Prod. Eng..

[8] Gellert R. Styropor and Lost Foam Casting Technology. Proceedings of the 4th annual EPC Conference.

[9] Alfredo D.D. Progress in the FIeld of Casting Quality Through Innovation and Evaluation of Tooling and Cluster Preparation Equipment. Proceedings of the 4th annual EPC Conference.

[10] Rodgers R.C. (1989). Navistar Restructures its Indianapolis Foundry for Global Competition. Foundry Manag. Technol..

[11] Sonnenberg F. (2003). Recent Innovations With EPS Lost Foam Beads. AFS Trans..

[12] Deev V.B., Ponomareva K.V., Kutsenko A.I., Prikhodko O.G., Smetanyuk S.V. (2017). Influence of melting conditions of aluminum alloys on the properties and quality of castings obtained by lost foam casting. Russ. J. Non-Ferr. Met..

[13] Jiang W., Li G., Fan Z., Wang L., Liu F. (2016). Investigation on the Interface Characteristics of Al/Mg Bimetallic Castings Processed by Lost Foam Casting. Met. Mater. Trans. A.

[14] Griffiths W.D., Ainsworth M.J. (2016). Instability of the Liquid Metal–Pattern Interface in the Lost Foam Casting of Aluminum Alloys. Met. Mater. Trans. A.

[15] Singh C.V., Warner D.H. (2010). Mechanisms of Guinier–Preston zone hardening in the thermal limit. Acta Mater..

[16] Duparc O.H. (2010). The Preston of the Guinier-Preston Zones. Guinier. Met. Mater. Trans. A.

[17] Tavitas-Medrano F.J., Mohamed A.M.A., Gruzleski J.E., Samuel F.H., Doty H.W. (2010). Precipitation-hardening in cast Al–Si–Cu–Mg alloys. J. Mater. Sci..

[18] Ibrahim M.F., Samuel A.M., Doty H.W., Samuel F.H. (2017). Effect of Aging Conditions on Precipitation Hardening in Al–Si–Mg and Al–Si–Cu–Mg Alloys. Inter. Met..

[19] Liu M., Fu H., Xu C., Xiao W., Peng Q., Yamagata H., Ma C. (2018). Precipitation kinetics and hardening mechanism in Al-Si solid solutions processed by high pressure solution treatment. Mater. Sci. Eng. A.

[20] Wilm A. (1911). Physikalisch-metallurgische Untersuchungen über magnesiumhaltige Aluminiumlegierungen. Métallurgie.

[21] Joseph S., Kumar S. (2015). Role of Si modification on the compressive flow behavior of Al–Si based alloy. Mater. Charact..

[22] Joseph S., Kumar S. (2013). A systematic investigation of fracture mechanisms in Al–Si based eutectic alloy—Effect of Si modification. Mater. Sci. Eng. A.

[23] Zhang W.X., Chen Y.Z., Zhou L., Zhao T.T., Wang W.Y., Liu F., Huang X.X. (2023). Simultaneous increase of tensile strength and ductility of Al-Si solid solution alloys: The effect of solute Si on work hardening and dislocation behaviors. Mater. Sci. Eng. A.

[24] Kim M.-J., Lee M.-G., Hariharan K., Hong S.-T., Choi I.-S., Kim D., Oh K.H., Han H.N. (2017). Electric current-assisted deformation behavior of Al-Mg-Si alloy under uniaxial tension. Int. J. Plast..

[25] Samuel A.M., Samuel F.H. (1992). Various aspects involved in the production of low-hydrogen aluminium castings. J. Mater. Sci..

[26] DiGiovanni M.T., de Menezes J.T.O., Cerri E., Castrodeza E.M. (2020). Influence of microstructure and porosity on the fracture toughness of Al-Si-Mg alloy. J. Mater. Res. Technol..

[27] Dash S.S., Chen D. (2023). A Review on Processing–Microstructure–Property Relationships of Al-Si Alloys: Recent Advances in Deformation Behavior. Metals.

[28] Robles Hernandez F.C., Herrera Ramírez J.M., Mackay R. (2017). Applications in the Automotive and Aerospace Industries. Al-Si Alloys.

[29] Pezda J., Jezierski J. (2020). Non-Standard T6 Heat Treatment of the Casting of the Combustion Engine Cylinder Head. Materials.

[30] Lu S.Z., Hellawell A. (1987). The mechanism of silicon modification in aluminumsilicon alloys: Impurity induced twinning. Metall. Mater. Trans. A.

[31] Makhlouf M., Guthy H. (2001). The aluminum-silicon eutectic reaction: Mechanisms and crystallography. J. Light Met..

[32] Timpel M., Wanderka N., Schlesiger R., Yamamoto T., Lazarev N., Isheim D., Schmitz G., Matsumura S., Banhart J. (2012). The role of strontium in modifying aluminium–silicon alloys. Acta Mater..

[33] Guzowski M.M., Sigworth G.K., Senter D.A. (1987). The role of boron in the grain refinement of aluminum with titanium. Metall. Trans. A.

[34] Gagnon D., Samuel A.M., Samuel F.H., Abdelaziz M.H., Doty H.W. (2021). Melt Treatment-Porosity Formation Relationship in Al-Si Cast Alloys. Casting Processes and Modelling of Metallic Materials.

[35] Abdelaziz M.H., Paradis M., Samuel A.M., Doty H.W., Samuel F.H. (2017). Effect of Aluminum Addition on the Microstructure, Tensile Properties, and Fractography of Cast Mg-Based Alloys. Adv. Mater. Sci. Eng..

[36] Gil-Santos A., Moelans N., Hort N., Van der Biest O. (2016). Identification and description of intermetallic compounds in Mg–Si–Sr cast and heat-treated alloys. J. Alloys Compd..

[37] Ibrahim M.F., Elgallad E.M., Valtierra S., Doty H.W., Samuel F.H. (2016). Metallurgical Parameters Controlling the Eutectic Silicon Charateristics in Be-Treated Al-Si-Mg Alloys. Materials.

[38] Van der Biest O., Gil-Santos A., Hort N., Schmid-Fetzer R., Moelans N., Orlov D., Joshi V., Solanki K., Neelameggham N. (2018). Study on Mg–Si–Sr Ternary Alloys for Biomedical Applications. Magnesium Technology 2018.

[39] Wu X., Zhang H., Ma Z., Tao T., Gui J., Song W., Yang B., Zhang H. (2019). Interactions between Fe-rich intermetallics and Mg-Si phase in Al-7Si-xMg alloys. J. Alloys Compd..

[40] Wu X.Y., Zhang H.R., Chen H.L., Jia L.N., Zhang H.R. (2017). Evolution of microstructure and mechanical properties of A356 aluminium alloy processed by hot spinning process. China Foundry.

[41] Chen R., Shi Y., Xu Q., Liu B.C. (2014). Effect of cooling rate on solidification parameters and microstructure of Al-7Si-0.3Mg-0.15Fe alloy. Trans. Nonferrous Met. Soc. China.

[42] Birol Y. (2012). Effect of silicon content in grain refining hypoeutectic Al–Si foundry alloys with boron and titanium additions. Mater. Sci. Technol..

[43] Chen Y., Pan Y., Lu T., Tao S., Wu J. (2014). Effects of combinative addition of lanthanum and boron on grain refinement of Al–Si casting alloys. Mater. Des..

[44] Sunitha K., Gurusami K. (2021). Study of Al-Si alloys grain refinement by inoculation. Mater. Today Proc..

[45] Zheng Q., Zhang B., Chen T., Wu J. (2024). Achieving superior grain refinement efficiency for Al–Si casting alloys through a novel Al–La–B grain refiner. J. Mater. Res. Technol..

[46] Park S.B. (2022). Heterogeneous nucleation models to predict grain size in solidification. Prog. Mater. Sci..

[47] Ammar H.R., Samuel A.M., Samuel F.H., Simielli E., Sigworth G.K., Lin J.C. (2012). Influence of aging parameters on the tensile properties and quality index of Al-9 pct Si-1.8 pct Cu-0.5 pct Mg 354-type casting alloys. Metall. Mater. Trans. A Phys. Metall. Mater. Sci..

[48] Zhao Y., Song D., Wang H., Li X., Chen L., Sun Z., Wang Z., Zhai T., Fu Y., Wang Y. (2022). Revealing the nucleation and growth mechanisms of Fe-rich phases in Al-Cu-Fe(-Si) alloys under the influence of Al-Ti-B. Intermetallics.

[49] Nowak M., Bolzoni L., Babu N.H. (2015). Grain refinement of A-Si alloys by Nb-B inoculation. Part I: Concept development and effect on binary alloys. Mater. Des..

[50] Bolzoni L., Nowak M., Babu N.H. (2015). Grain refinement of Al-Si alloys by Nb-B inoculation. Part II: Application to commercial alloys. Mater. Des..

[51] Samuel E., Samuel A.M., Songmene V., Samuel F.H. (2023). A review on the analysis of thermal and thermodynamic aspects of grain refinement of aluminum-silicon-based alloys. Materials.

[52] Srirangam P., Chattopadhyay S., Bhattacharya A., Nag S., Kaduk J., Shankar S., Banerjee R., Shibata T. (2014). Probing the local atomic structure of Sr-modified Al–Si alloys. Acta Mater..

[53] Ganesh M.R.S., Reghunath N., Levin M.J., Prasad A., Doondi S., Shankar K.V. (2022). Strontium in Al–Si–Mg Alloy: A Review. Met. Mater. Int..

[54] Fracchia E., Gobber F.S., Rosso M. (2021). Effect of Alloying Elements on the Sr Modification of Al-Si Cast Alloys. Metals.

[55] Li P., Kandalova E.G., Nikitin V.I. (2005). Grain refining performance of Al-Ti master alloys with different microstructures. Mater. Lett..

[56] Sumalatha C., Rao P.C.S., Rao V.S., Deepak M.S.K. (2022). Effect of grain refiner, modifier and graphene on the mechanical properties of hyper eutectic Al-Si alloys by experimental and numerical investigation. Mater. Today Proc..

[57] Okayasu M., Yoshida S. (2014). Influence of solidification rate on material properties of cast aluminium alloys based on Al–Si–Cu and Al–Si–Mg. Int. J. Cast Met. Res..

[58] Zhu B., Leisner P., Seifeddine S., Jarfors A.E. (2016). Influence of Si and cooling rate on microstructure and mechanical properties of Al-Si-Mg cast alloys. Surf. Interface Anal..

